# Environmental Impact of the Presence, Distribution, and Use of Artificial Sweeteners as Emerging Sources of Pollution

**DOI:** 10.1155/2021/6624569

**Published:** 2021-04-14

**Authors:** Ab Qayoom Naik, Tabassum Zafar, Vinoy Kumar Shrivastava

**Affiliations:** Laboratory of Endocrinology, Department of Biosciences, Barkatullah University, Bhopal 462026, MP, India

## Abstract

Artificial sweeteners are posing a new threat to the environment. The water ecosystem is the primary recipient of these emerging contaminants. Once ingested, sufficient amount of these artificial sweeteners escape unchanged from the human body and are added to the environment. However, some are added in the form of their breakdown products through excretion. Artificial sweeteners are resistant to wastewater treatment processes and are therefore continuously introduced into the water environments. However, the environmental behavior, fate, and long-term ecotoxicological contributions of artificial sweeteners in our water resources still remain largely unknown. Some artificial sweeteners like saccharin are used as a food additive in animal feeds. It also forms the degradation product of the sulfonylurea herbicides. All artificial sweeteners enter into the wastewater treatment plants from the industries and households. From the effluents, they finally reside into the receiving environmental bodies including wastewaters, groundwaters, and surface waters. The global production of these sweeteners is several hundred tons annually and is continuously being added into the environment.

## 1. Introduction

Artificial sweeteners (ASs) are the food additives used in thousands of food products throughout the world. Among many food products containing ASs are food and beverages, desserts, chewing gums, pastries, and breads especially for diabetic and/or obese people. Besides, these are also used in other personal care and pharmaceutical products [1–4] such as toothpastes and cough syrups. The most popular ASs exhibiting countless food chemistry applications are saccharin (SAC), cyclamate (CYC), aspartame (ASP), acesulfame (ACE), sucralose (SUC), alitame (ALT), neotame (NEO), and neohesperidine dihydrochalcone (NSDH) [5–7]. The use of ASs in drugs and sanitary products has also been confirmed [4, 8].

ASs are primarily used for the processing of low-calorie foods without sugar in the food industry. These sweeteners are widely used in human diet as they do not induce any glycemic effect/insulin reaction once ingested. Besides, they do not release any calories and do not have any adverse effect on the dental plaque microflora, unlike sugar [6]. CYC is currently the most developed artificial sweetener among sulfamates, followed closely by SAC [8]. In diet soft drinks, ASP and ACE-K-k are the leading brands, while SUC has been the leader in the main market for tabletop sweeteners. A class of pollutants that have entered the environment for years and have not been thoroughly studied so far in terms of occurrence, environmental fate, and toxicity assessment and/or protected by current worldwide regulations are classified as “emerging pollutants” or “emerging contaminants” [9–11]. ASs are a newly found class of environmental contaminant because of their prolonged existence and universal occurrence in various aquatic ecosystems.

It is believed that the number of emerging pollutants is more than 700 that are being added to the environment on daily basis [12]. In view of the growing pollution stress, these emerging pollutants should be explored and addressed in a systemic manner to understand their potential risk to the environment and human health [11]. The global consumption of ASs is reported to be more than 159,000 metric tons. This amounts to the market value of USD $2 billion. China is currently the leading country consuming the most ASs (32%), followed by Asia/Oceania (23%), United States (23%), Europe (12%), and Africa (7%) [13]. Global use of nonnutritive ASs in food and beverage products indicated ASP has the highest use with 18.5 thousand metric tons, followed by SAC (9.7 thousand metric tons), ACE-K (6.8 thousand metric tons), and SUC (3.3 thousand metric tons) [14]. An extremely high concentration of ASP is used in the food industry (as much as 5602 mg/kg) in Korea [15]. ASP represents the largest artificial sweetener product segment globally; it is the most popular artificial sweetener in the U.S. and is used in more than 6000 food products [16].

Although they are found in very low concentrations, emerging contaminants have a great potential to trigger adverse human and the environmental effects as reported by some researchers in *in vivo* and/or *in vitro* laboratory studies at very low concentrations. Since they are found in very low concentrations (micrograms or milligrams per kg_dw_) in the environment, emerging contaminants are also known as trace pollutants [17, 18]. Therefore, these contaminants are the focus of several studies due to their potential environmental impacts attributed to their toxicity for aquatic and terrestrial organisms [18–21]. A significant proportion of SAC, CYC, ACE-K-k, and SUC move from the human body in an unchanged form following ingestion [22, 23], while ASP, ALT, NEO, and NSHD are removed to a greater degree [24]. The analysis of the ASs in wastewater influents and effluents was detected only in five (ACE-K, CYC, SAC, and SUC) out of seven ASs positively. Wastewater samples (downstream of wastewater effluents) from three different locations were analysed for the presence of ASP, SAC, and SUC in USA. Of the five water samples analysed, detectable concentrations of SUC were found in all samples at 0.8 and 1.8 *μ*g/L. However, SAC was detected at one location only at a concentration of 5 *μ*g/L [25, 26]. Estimated 30% of the samples had detectable amounts of one or more ASs, suggesting the presence of water derived from septic system effluent. Likewise, 2.0 to 4.7 percent of groundwater seeps had a septic effluent contribution of 1% or more [27]. A remarkable influence is exhibited by the type of eluents and the pH on the ASs recovery using solid-phase extraction cartridge (SPE) [4].

Maximum limit of the artificial sweeteners as defined by the Ministry of Health and family Welfare, Food Safety and Standards Authority of India, [28] is given below (Table 1).

## 2. Studies Search and Selection Criteria

Literature search was performed by searching databases like PubMed, PubMed Central, Research Gate, Google Scholar, Medline, and Science Direct. The research articles based on the studies are related with nonnutritive ASs and their impact in environment. The search terms which were used during the literature search include the presence, source, route of transfer, and environmental impact and estimation techniques of the ASs. Studies including the detection techniques and regional studies were also included in the present review. The focus of the review is ASs as emerging contaminants.

### 2.1. Environmental Impact of the ASs

ASs have been reported to have negative health effects in both humans and aquatic organisms. They were previously unrecognized in terms of their health effects due to lack of standards and guidelines for their environmental monitoring and have only recently been identified as potential environmental contaminants [29]. The presence of ASs was initially detected in water and soil [30]. A considerable amount of these ASs were reported to remain unchanged and poorly absorbed [31]. The discharged ASs from different sources make their way to the wastewater treatment plants (WWTPs). However, most of the ASs escape even the most efficient WWTPs. Meanwhile, treated effluents from these WWTPs are believed to be major point sources of ASs which ultimately find their way into the environment [32]. The concentrations of ASs in wastewater effluents were found to have no effect of the wastewater treatment even by using nanograms per liter [32, 33].

ASP is hydrolyzed to aspartylphenylalanine below the pH of 3 and above the pH of 6, and it gets transformed into 5-benzyl-3,6-dioxo-2 piperazine acetic acid [34, 35]. ALT is soluble in water (approx. 13.1% w/v at 25°C) and is relatively stable to heat because of its unique amide group. Upon hydrolysis, ALT is converted to alanine amide, aspartic acid, and b-aspartic isomer, and in the human body, the *N*-glucuronide is the major metabolic product [36]. NEO is an *N*-substituted ASP derivative [37], with its major degradation product being de-esterified NEO (WHO Food Additive Series 52: NEO (http://www.inchem.org/documents). Neohesperidin dihydrochalcone is converted by humans anoxically to 3-(3-hydroxy-4-methoxyphenyl)propionic acid or 3-(3,4-dihydroxyphenyl)propionic acid [38].

Thus, the major metabolites of ASP, NEO, ALT, and NSHD should be expected to be present in the aquatic environment instead of their parent compounds. SAC is one of the oldest ASs. Its commercial production started in 1878. It is not metabolized by the body and is excreted unchanged in the urine once ingested [39], and the unabsorbed portion is excreted with faeces [40]. SAC is found in groundwater due to application of fertilizers in agriculture, degradation of sulfonylurea herbicides, old landfills, irrigation, soil water management, use of sludge as a fertilizer, and leaks in the ducts. Municipal wastewater and sewage are also found to contain SAC. SAC is used as a galvanic brightener in industry in smaller amounts [40]. SUC has been detected in coastal and sea waters. It was further reported that concentrations up to 0.21 *μ*g/l of SAC were found in surface waters in China [41]. ACE-K-k, SAC, SUC, and CYC which are used as sugar substitutes are the most detected food additives/sweeteners in soils and groundwater [17, 42].

However, many ASs are not degradable and could also be introduced into the soil environment [43]. ASP is a dipeptide methyl ester of l-aspartyl-l-phenylalanine. For certain foods which are stored for longer durations, such as carbonated and still beverages, the stability of ASP in aqueous media is not satisfactory. As a dipeptide ester, ASP undergoes both hydrolysis and cyclization reactions. Under acidic conditions, hydrolysis of the ester and amide bonds is favored, resulting in the formation of its constituent amino acids with a concomitant loss of sweetness. More neutral and alkaline conditions are favorable for the cyclization of ASP to the corresponding diketopiperazine [44]. The physical and chemical properties and molecular structure of some of the ASs are given in Tables 2 and 3 respectively.

ASs are recognized as a new class of environmental contaminants due to their extreme persistence and ubiquitous nature. The continuous introduction of ASs into the aquatic environments is attributed to their resistant behavior to wastewater treatment processes. However, behavior, fate, and long-term ecotoxicological contribution of ASs in water resources is at a large still unknown. The study of geographical/seasonal/hydrological interactions of ACE-K, CYC, SAC, and SUC in an open coast system at an estuarine/marine junction revealed higher occurrence of ACE-K (seasonal average: 0.22 *μ*g L (−1)) and SUC (0.05 *μ*g L (−1)) was noted in summer, while SAC (0.11 *μ*g L (−1)) and CYC (0.10 *μ*g L (−1)) were predominantly detected in winter, suggesting a strong connection with the variable chemical resistance among different sweeteners [30, 51]. The formation of new photo by-products under prolonged UV irradiation is highly viable in ACE-K and SUC compounds as were revealed by UPLC-ESI/MS degradation profile. Photodegradation results revealed that phototoxicity of ACE-K products may affect aquatic ecosystems [30, 59].

A study on estimated inputs from agriculture and households, degradation, and leaching to groundwater revealed that considerable concentration of SAC may end up in soils through liquid manure. SAC, found in piglet feed as an additive, is largely excreted. SAC may thus end up in soils in considerable quantities with manure up to a concentration of 12 mg/l and is stable for two months of storage. It was also observed that SAC is a soil metabolite of certain sulfonylurea herbicides. Meanwhile, ASs may get into the soil through irrigation with wastewater-polluted surface water and fertilization with sewage sludge (1–43 *μ*g/L) or through leaky sewers. The soil incubation experiments revealed that CYC, SAC, ACE-K, and SUC were degraded with half-lives of 0.4–6 d, 3–12 d, 3–49 d, and 8–124 d, respectively. The study of the relative importance of entry pathways to soils, degradation, and leaching to groundwater revealed presence of SAC in groundwater (0.26 *μ*g/L) is due to the application of manure. On the contrary, concentration of ACE-K (up to 5 *μ*g/L) may be due to the infiltration of wastewater-polluted surface waters through stream beds [23, 42, 60].

The widespread presence of ASs in various environmental samples and their efficient recovery and accurate determination in environments depends on the various external factors including solid-phase extraction (SPE) cartridges, buffers and pH, matrix effects, and sample stability. The mainstream method of determination employed is SPE with LC-ESI-MS. The ASs, widely found in various environmental media, are ACE-K and SUC, and their investigated concentrations were found in the order of wastewater treatment plants (WWTPs) influent > WWTPs effluent > surface water > groundwater > drinking water; and atmosphere > soil. AS levels exhibit significant differences among different regions [61, 62].

ASs have been identified as emerging environmental pollutants and can be found in receiving waters, i.e., surface waters, groundwater aquifers, and drinking waters. Relative toxicity of ASs was studied by bioluminescence activity assay (using genetically modified bioluminescent bacteria from *E. coli*). Toxic effects were observed as the bacteria were exposed to certain concentrations of the ASs. In the bioluminescence activity assay, two toxicity response patterns were observed, namely, the induction and inhibition of the bioluminescent signal. An inhibition response pattern was observed in the response of SUC and NEO. Besides, the tested bioluminescent bacterial panel can potentially be used for detecting ASs in the environment, using a specific mode-of-action pattern [63]. SUC is increasingly being used as what scientists call a “tracer”—a substance that can help determine where contamination comes from. This capacity is essential for maintaining the quality of water, both in surface waters and in the supply of drinking water (https://www.scientificamerican.com).

Emerging pollutants are primarily of industrial origin and medical discharge and are translocated to soils, from soils to plants, and finally to consumers in the form of vegetables, fruits, etc. irrigated by treated wastewater or untreated surface water. Steroidal estrogens, bromofen, ibuprofen, caffeine, methyl dihydrojasmonate, etc. are some of the emerging pollutants other than ASs. The concentration of these pollutants in agricultural waters used for irrigation has been reported to range from 10 to 500 ng/L. The concentration of these pollutants in crops was found to be ranging from 1 to 7500 ng/kg [64]. Groundwater contamination with ASs has a number of different direct and indirect routes including percolation of treated wastewater in soil aquifer treatment, infiltration of wastewater-influenced surface waters, landfill leachate, municipal wastewater reservoirs, septic systems, and percolation of manure on agricultural land [8, 23, 65, 66].

ASs including ASP and SAC at the limit concentration of 100 mg/l was tested for their impact on model species like *Lemna minor, Sinapis alba, Daphnia magna, Enchytraeus crypticus, Desmodesmus subspicatus*, and *Lactuca sativa*. The study revealed statistically negative effects on *Lemna minor*, while as both ASP and SAC had negative reproductive effects on enchytraeids [67].

### 2.2. Source and Pathways of Emerging Contaminants

**Figure 1:**
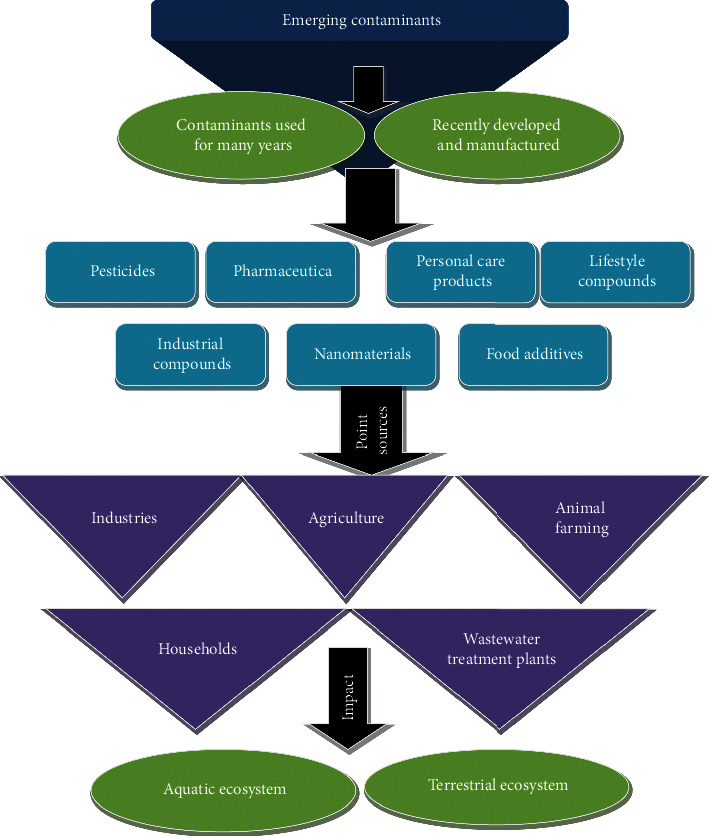
Flow chart showing emerging contaminants, sources, and their end points.

Input of contaminants into the soil and water is largely due to anthropogenic activities [68, 69] (Figure 1). Pond sources of contamination of soil, surface, and groundwater resources include discrete locations, municipal sewages, water treatment plants, accidental leaks, and landfills. Nonpoint or diffuse sources of contamination are agriculture and animal farming, industrial contaminants, exhaust gases (both industrial and vehicular), recycling, and untreated wastewater [70–75]. Emerging contaminants find their way into the soil and water environments through a source-pathway receptor model, wherein a pathway is necessary for the transport of these contaminants into these resources. Soils and water form the pathway, while living organisms (both aquatic and soil) are considered as receptors. The notable pathways include intentional disposal, landfill leachates, sewage leaks, irrigation with treated/untreated wastewaters, and animal farms [17, 74, 76–78]. Others include atmospheric air affecting plants and animals, deposition of contaminants on plant surfaces or soil, and gradual accumulation. These contaminants may persist for many years or spread through the soil profile and finally end up into the groundwater, besides incidental and structural spills and diffuse emissions [9].

The high detection frequency of ASs in various environmental media has created great concern. Studies on ASs ecotoxicity and possible elimination routes in the environment revealed that negative impacts of ASs are more severe than expected, and the focus should be on the chronic and metabolic toxicities of ASs. Among the various ASs, CYC and SAC are easily removed, while SUC and ACE-K are generally persistent. The potential for microbial degradation of persistent ASs was reported in some regions, but clarification of the underlying mechanisms is necessary to increase the likelihood of using this approach in wide applications with a satisfactory performance [79, 80]. Emerging contaminants (pesticides and their breakdown products, pharmaceuticals, personal and house care products, lifestyle compounds, food additives, industrial products and wastes, and nanomaterials) have become a concern for the environment. Cumulative use of these products in industries, agriculture, domestic use, and healthcare services has led to their appearance in significant amounts in soils, surface, and groundwater resources with unpredictable consequences. Toxicity and bioaccumulation data of these emerging contaminants are limited [81].

The presence of various emerging contaminants including pharmaceuticals and personal care products (PPCPs) in groundwater is mainly attributed to anthropogenic activities as most of the compounds detected in the water samples are synthetic products, except caffeine [82]. ASs and PPCPs find their way into the groundwater from different sources and pathways including household PPCPs, industrial and hospital wastewater, drugs, and AS containing foods. The synthetic products or their breakdown compounds are added to landfills, septic system, STP effluent, sewer, soil, and surface water through livestock excreta, solid waste, surface water, toilets, and or sinks. These compounds are finally added to the groundwater in varied concentrations due to the addition of PPCPs containing sewage waters, leakage or managed aquifer recharge from sewer, sewage treatment plants, and septic system. A number of compounds including antibiotics, anti-inflammatories, lipid regulators, psychiatric drugs, stimulants, insect repellants, and sunscreen agents have been reported in groundwater in different concentrations [83].

A number of policies framed for the protection of the groundwater have a continuous challenge with the addition of bulk (700 approx.) emerging contaminants from various points and diffuse sources besides conventional pollutants [82].

### 2.3. Ecotoxicity of the ASs

The worldwide environmental distribution of ASs has prompted researchers towards the assessment of the ecotoxicological impact of these ASs. Depending on the presence, persistence, and bioaccumulation potential, ASs have varied toxic effects in the ecosystem, plant growth, and aquatic organisms [24]. SUC has been reported to affect the physiology and locomotive behavior of *Daphnia magna* at 0.0001–5 mg/L. Altered swimming height and increased swimming speed were observed in this planktonic crustacean. Besides, the time to reach food and shelter was found to be prolonged in Gammarid [84]. However, *Daphnia magna* and Mysida shrimp *(Americamysis bahia)* were reported to have no effect of SUC at ≤1800 mg/L and ≤93 mg/L of SUC, respectively, on their survival, growth, and reproduction [85]. Short-term effects of SUC on egg production, hatching rate, food intake, and mortality of two species of Arctic copepods at concentrations ranging from 0 to 50,000 ng/L showed nonsignificant effects on these parameters [86]. A recent study has reported that ASP (100 mg/kg) was toxic to *Lemna minor, Sinapis alba, Daphnia magna, Enchytraeus crypticus, Desmodesmus subspicatus,* and *Lactuca sativa* while both SAC and ASP (100 mg/kg) disrupted the reproduction of Enchytraeidae [67].

A study investigated the relative toxicity of the ASs using genetically modified bioluminescent bacteria from *E. coli*. The bioluminescent bacteria luminesce when they detect toxicants. The bacteria were exposed to varied concentrations of the ASs to study the toxic effects. In this assay, two toxicity response patterns were reported, namely, the induction and inhibition of the bioluminescent signal. An inhibition response pattern may be observed in the response of SUC in all the tested strains: TV1061 (MLIC = 1 mg/mL), DPD2544 (MLIC = 50 mg/mL), and DPD2794 (MLIC = 100 mg/mL). It is also observed in NEO in the DPD2544 (MLIC = 2 mg/mL) strain. However, the induction response pattern may be observed in its response in SAC in TV1061 (MLIndC = 5 mg/mL) and DPD2794 (MLIndC = 5 mg/mL) strains, ASP in DPD2794 (MLIndC = 4 mg/mL) strain, and ACE-K in DPD2794 (MLIndC = 10 mg/mL) strain [63].

The increased toxicity of ACE-K and SUC intermediates to living organisms following photolysis or electrolysis has also been documented in several other studies [87, 88]. The increased ecotoxicity of ACE-K after UV irradiance was found to be induced by the accumulation of OH• which caused a strong oxidative status even at a concentration of 0.1 mg/L in the liver of fish [89].

A study indicated that SUC disrupted the gene responsible for the absorption of sucrose from sugarcane, thereby inhibiting the absorption and transportation of sucrose within plants [90]. A strong positive association was observed between the concentration of SUC (0.0001–5 mg/L) and neurological and oxidative changes that could cause sublethal effects in Daphnia [91]. In addition, at environmentally appropriate concentrations (0.05 and 155 *μ*g/L) with various exposure periods (12, 24, 48, 72, and 96 h), the possible SUC-induced toxicological hazard to *Cyprinus carpio* was also assessed. The results indicated that the content of lipid peroxidation, hydroperoxide, and protein carbonyl and the activity of antioxidant enzymes in the tested species especially in the gills, brain, and muscles were significantly increased [92]. SAC and CYC have also recently been found to exhibit plant cytotoxic and mutagenic (*Allium cepa*) effects at permitted levels [93].

In addition, it should be noted that while the parent ASs have been shown to be minimally harmful to aquatic habitats, they can potentially turn through different pathways into more toxic metabolites. The acute toxicity calculation of artificial sweetener metabolites showed that the acute inhibition effect of ACE-K-k in *V. fischeri* was significantly amplified to EC50 = 125.5 mg/L after photo treatment, with a measurable magnification factor of 575. Further study revealed that a calculated increase in toxicity (above 99.5 percent) was due to metabolite accumulation. This result is unusual since the enhancement factor for other persistent organic contaminants is rarely more than 20 times higher, illustrating the great concerns about ASs. Studies have found that the hazardous amplification of SUC ranged from 2670 mg/L to 156.2 mg/L with a detectable magnification factor of 16.5. (close to the “harmful” criterion of SUC of 100 mg/L). Considering the toxicity results given the more persistent and toxic by-products, ACE-K appeared to have a long-term ecological effect than SUC [30].

Furthermore, since a variety of contaminants can coexist in the environment, the toxic effects of AS mixtures with other chemicals in the environment should also be considered. In the order of classification of SAC > caffeine > ASP > SUC, ASs can affect the heart rate, eye density, and body length of the fish, and a cumulative effect combined with caffeine was observed [94]. Besides, some findings may consider that, at current environmental levels, ASs show reasonably low acute toxicity to species [86, 95].

Significant advancement in the analysis and detection has helped in discovery and quantification of emerging contaminants not only in living beings but also in diverse environmental substances. However, little data are available on the adverse effects of environmental exposure on the general population [69].

## 3. Conclusion

Artificial sweeteners being discussed for their positive and negative health implications are yet to be solved. A new addition into this debate is the negative impact of the ASs on the environment. As these are called as emerging contaminants, these ecotoxic compounds have added to the already extreme load of other pollutants. A two-way tackling methodology is required to contain the negative impacts of these emerging contaminants which are affecting both the human health as well as the health of the ecosystem. These observations may also indicate that the concentrations of SUC detected in the environment were well below of what is required to elicit effects in freshwater or marine invertebrates. However, it is also important to mention that, of the many ASs being used worldwide, the presence and concentration and ecotoxicity vary from one ecosystem to another. Therefore, observations made a few years back may not be the same at present as the number and bulk both increase at a very fast rate along with other environmental factors and pollutants. The growing evidence of the increased concentration and ecotoxicity of the ASs in the environment demands complete risk assessment, estimation and ecotoxicity tests, and elimination of these emerging contaminants. The review highlights the ecological impact of the ASs and their breakdown products which have recently been classified as emerging contaminants. The presence of the ASs and their degradation products in aquatic systems is a new threat to the aquatic life due to their presence in significant concentration. Besides testing, estimation techniques, sources, and transformation pathways, the review is expected to benefit the scientific community in future prospects for environment safety assessment.

## Figures and Tables

**Table 1:** Maximum permissible limit of various artificial sweeteners approved by FSSAI.

Artificial sweetener	Food article	Maximum limit (ppm)
Sodium saccharin	Carbonated water	100
	Soft drink concentrate	100

Aspartame	Carbonated water	700
	Soft drink concentrate	700

Sucralose	Carbonated water	300
	Soft drink concentrate	300

Acesulfame-K	Carbonated water	300
	Soft drink concentrate	300

Neotame	Carbonated water	33
	Soft drink concentrate	33

*Source.* Ministry of Health and Family Welfare, Food Standards Authority of India; Notification No. 01 of August 2011.

**Table 2:** Physical and chemical characteristics of most widely used artificial sweeteners and their main markets worldwide.

Artificial sweetener	Brand/trade name	Main market	Chemical formula	ADI (g/kg/day)	Sweetening potential	Uses	Molar mass (g/mol)	Density (g/cm^3^)	Year of approval	Reference
ASP	NutraSweet Equal	North America, Europe, Asia	C_14_H_18_N_2_O_5_	40	200	Foods and beverages, pharmaceuticals, etc.	294.3	1.347	1981	Whitehouse et al., 2008 [45]; Fenge et al., 2013 [46]; Abu-Reidah [47]

SAC	Sweet'N Low, Sweet Twin, Necta Sweet	America, Europe, Asia	C_7_H_5_NO_3_	5	300	Soft drinks, tabletop sweeteners, and desserts	183.2	0.83	1985	Walter [46]; Feng et al., 2013 [48]; BeMiller [49]

ACE	Sunett Sweet One	North America, Europe, Asia	C_4_H_4_KNO_4_S	15	200	Table-top sweeteners, beverages, dairy products, confectionery, oral hygiene products, and pharmaceuticals	163.15	1.83	1988	Kuhn [50]; Lange et al. [51]; Zeece [52]

NEO	Neotame	America	C_20_H_24_N_2_O_5_	0.3	7000	Carbonated soft drinks, yogurts, cakes, drink powders, etc.	378.46	1.13	2002	Nabors [54]; Lange et al. [51]; Tiefenb-acher [53]

SUC	Splenda	North America	C_12_H_19_Cl_3_O_8_	5	6000	Diet foods and beverages	397.64	1.69	1998	Spillane [47]; Feng et al., 2013 [48]; Hughes and Dean [56]

ALT	Aclame	Australia, Mexico, New Zealand, China	C_14_H_25_N_3_O_4_S	1	2000	Bakery products, snack foods, candies and confectionery, ice cream, and frozen dairy products	331.43		Not approved by FDA, thus not used in USA and EU	Feng 2016 et al., 2013 [48]; Tiefenb-acher [53]; BeMiller [49]

CYC	Twin Sweet	Europe, Asia	C_6_H_12_NNaO_3_S	7	30	Baked goods, confections, desserts, soft drinks, preserves, and salad dressings	201.22	0.7	1984 banned in USA since 1970	Lange et al., 2012 [51]; Chakrab-orty and Das, 2016 [57]

NHDC		Europe, Japan	C_28_H_36_O_15_	5	1900	Flavoring agent or adjuvant, foods including condiments and seasonings, beverage, and pharmaceuticals	612.6	1.6	1994	Lange et al. [51]; Ashurst et al. [58]

**Table 3:** Molecular structures of the most widely used artificial sweeteners and a new class of emerging pollutants.

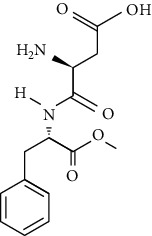	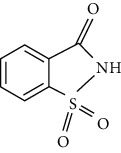	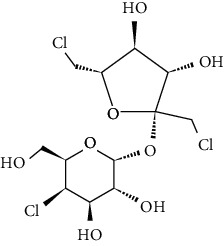
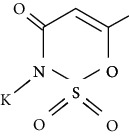	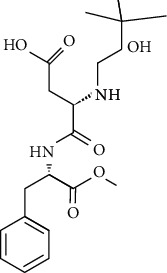	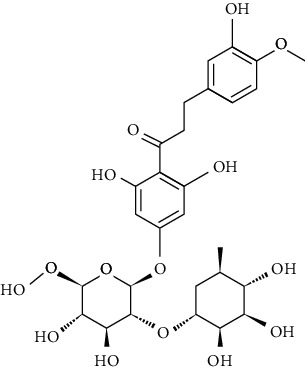
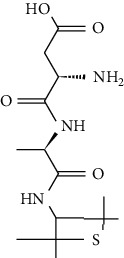		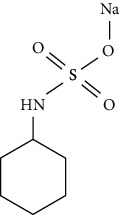

Molecular structures were redrawn using Chemdraw®.

## Data Availability

Data are available within the article and its supplementary materials
